# Deep asymmetric extraction and aggregation for infrared small target detection

**DOI:** 10.1038/s41598-023-48341-9

**Published:** 2023-11-29

**Authors:** Zhongwu Lin, Yuhao Ma, Ruixing Ming, Guohui Yao, Zhuo Lei, Qinghui Zhou, Min Huang

**Affiliations:** School of Statistics and Mathematics, Zhejiang Gongshang University, Qiantang District, Hangzhou, 310018 China

**Keywords:** Computer science, Optics and photonics

## Abstract

Infrared small target detection is widely applied in military and civilian fields. Due to the small size of infrared targets, textural detail is missing. Common target detection methods extract semantic feature by narrowing down the feature map several times, which may lead to the small targets lost in deep layers and are not effective for infrared small target detection. To solve this problem, we propose a novel network called deep asymmetric extraction and aggregation. The network mainly consists of two processes - the vertical feature extraction and the horizontal feature aggregation, both of which are enhanced by an asymmetric attention mechanism. In the vertical process, the use of asymmetric attention mechanism combined with the reduction of down-sampling makes the small target better retained in the deep layers. Then through the horizontal process, shallow spatial feature and deep semantic feature are aggregated to further highlight the small targets while suppressing background noise. Experiments on the public datasets NUAA-SISRT, NUDT-SISRT and MDvsFA-cGan show that our proposed network outperforms the state-of-the-art methods in terms of detection accuracy and parameter efficiency.

## Introduction

Infrared small target detection has always been an important issue for infrared image. Unlike visible imaging, infrared imaging captures the intensity of infrared radiation emitted by objects, which can penetrate obstacles such as cloud and fog. Therefore, infrared imaging has the advantage of being independent of light condition and weather change. It is widely used in military and civilian fields such as military early warning system and nighttime search and rescue mission. However, these applications have a wide field of view and long observation distance, so that a target occupies only a small number of pixels in the infrared images^1^. Infrared small target lacks textural detail and contour shape feature. Common target detection methods are not applicable to small infrared target, so new detection methods are in need for infrared small target detection.

Most of the traditional model-driven methods for infrared small target detection are based on the differences between target and background, and transform the problem into the detection of outlier point. These methods only consider shallow spatial feature and lack semantic distinction between target and background noise, resulting in a large number of false alarms and miss detections.

Deep learning, a data-driven approach, is based on learning target feature through training. Convolutional neural networks (CNNs) are effective at extracting high-level semantic feature that improve the capacity to distinguish target from background noise. Ordinary CNNs extract advanced semantic feature by narrowing the feature map through gradual down-sampling operations^2^. However, in the case of small infrared target, the background feature can easily overwhelm small target during down-sampling. Thus we need to improve the network to make the targets stand out on the background, and reduce the number of down-sampling operations as possible. One way to achieve that is to add attention mechanism to the coding process and adaptively highlight the target through the network training process.

Specifically, we propose a network called deep asymmetric extraction and aggregation (DAEA). The DAEA network mainly consists of two types of process, the vertical feature extraction and the horizontal feature aggregation, both of which are enhanced by an asymmetric attention mechanism. Many current attention mechanisms are mainly classified as spatial attention and channel attention, or alternating between the two. However, due to the specificity of infrared small targets, the network is required to pay attention to both semantic information and spatial details of the small targets. Therefore, we propose an asymmetric attention mechanism (AAM) to enable the network to pay attention to both semantic information and spatial details of infrared small targets. The basic idea of AAM is that by combining global and local, spatial and semantic features, the enhancement of small target features makes it not easy to be lost in the deep layers. Through the vertical process, both global and local information are focused on, thus retaining small targets in deep layers. The shallow spatial features and deep semantic features are then iteratively aggregated by horizontal processing to further highlight small targets while suppressing background noise. Horizontal processing using iterative aggregation^3^ can reduce the influence of shallow features on image prediction.

Due to the generality of the AAM idea, which is an abstract representation of a combination of spatial and channel attention mechanisms, there can be a variety of concrete implementations in different application scenarios. In this paper, we adopt shuffle attention (SA)^4^ and asymmetric context modulation (ACM)^5^ as the asymmetric attention modules in our vertical process and horizontal process, respectively. The final feature map is then used as input to the prediction module for image segmentation. Experiments on the public datasets NUAA-SISRT, NUDT-SISRT and MDvsFAcGan show that DAEA outperforms the SOTA methods in terms of both detection accuracy and parameter efficiency. Finally, ablation experiments were performed to investigate the effectiveness and asymmetric advantages of AAM for infrared small target detection.

Our main contributions are as follows:

1. A novel DAEA network is proposed for infrared small target detection. DAEA distinguishes small target from background noise more effectively due to full use of shallow spatial feature and deep semantic feature by iterative aggregation. Using iterative aggregation can reduce the influence of shallow features on image prediction.
2. Semantic feature is more relevant to small target because it is better retained on deep layers, which is achieved by fused global and local attention and reduced number of down-sampling operations. This, in turn, makes the following feature aggregation more meaningful.
3. Experiments on the public datasets NUAA-SISRT, NUDT-SISRT and MDvsFA-cGAN demonstrate the superiority of our proposed network, outperforming the current SOTA methods by more accurate detection with less parameters.

## Related work

There are two main types of methods for the problem of infrared small target detection, one is multi-frame detection^6^, and the other is single-frame detection. The former generally uses a set of consecutive sequential images to detect the continuity of target in adjacent frames assuming that the background of the adjacent frames is stationary. However, in real scenario the infrared sensor needs to constantly adjust its angle in order to be able to capture fast moving object, which leads to the assumption that the background is stationary no longer satisfied^7^. Moreover, the efficiency of multi-frame detection is low and cannot meet the task of real-time detection. Therefore, single frame detection has attracted more attention.

For single-frame infrared small target detection, approaches are mainly classified into model-driven and data-driven ones. Most model-driven approaches convert the problem to outlier detection^8,9^, highlighting small target by measuring the discontinuity between them and the background. They include filter-based methods^10,11^, local contrast-based methods^12,13^, and low-rank-based methods^14,15^, which mostly use local mean or maximum gray level as features to manually set thresholds for target segmentation. These models do not need to be trained and follow the predefined process and hyper-parameters^16^ to achieve detection results. However, in practice, it is found that the biggest problem with these methods is that it is difficult to achieve good detection results using fixed hyper-parameters in the face of scene changes. At the same time, it is difficult to distinguish between background noise and target, resulting in a large number of false detections. Small target with different size and insignificant feature are easily overwhelmed by the background leading to inaccurate detection of target.

Deep learning is based on data-driven learning of target feature, which has been quite effective in the field of computer vision in recent years. Thanks to the powerful fitting ability of CNNs and the large amount of data labeling work, it is practical for CNNs to learn target feature accurately. Data-driven approaches show superior performance compared to traditional model-driven approaches. Liu et al.^17^ first used a target detection framework for detecting small infrared target, and their network was a 5-layer multilayer perceptual neural network. Zhao et al.^18^ proposed a generative adversarial network (GAN) based detection model for infrared small target detection. Wang et al.^19^ used conditional generative adversarial network (CGAN), which treated miss detection and false alarm as two opposing problems and trained the network to make a trade-off between the two metrics.

Image segmentation approaches have also received much attention, especially the extensive use of U-Net^20^ for medical image segmentation, which is now applied to infrared small target detection. Zhao et al.^21^ used U-Net combined with a semantic constraint module to achieve semantic segmentation of infrared small target. Dai et al.^5^ designed an asymmetric contextual module for image segmentation network, the network fuses high-level and low-level features to extract rich semantic information and spatial detail. Dai et al.^12^ designed a trainable attentional local contrast network in combination with a model-driven approach in subsequent network improvements. Li et al.^22^ designed a tri-direction dense nested interactive module and incorporated an attention mechanism, cascaded channels and a spatial attention module to set multiple nodes interconnected in the encoding and decoding paths to achieve repetitive feature fusion and enhancement. Although these networks have improved in performance but still cannot solve the problem of small target lost in the deep network coding process. How to keep the small target on the deep layers is the key to solve the problem of infrared small target detection.

## Methods

### Network architecture

**Figure 1:**
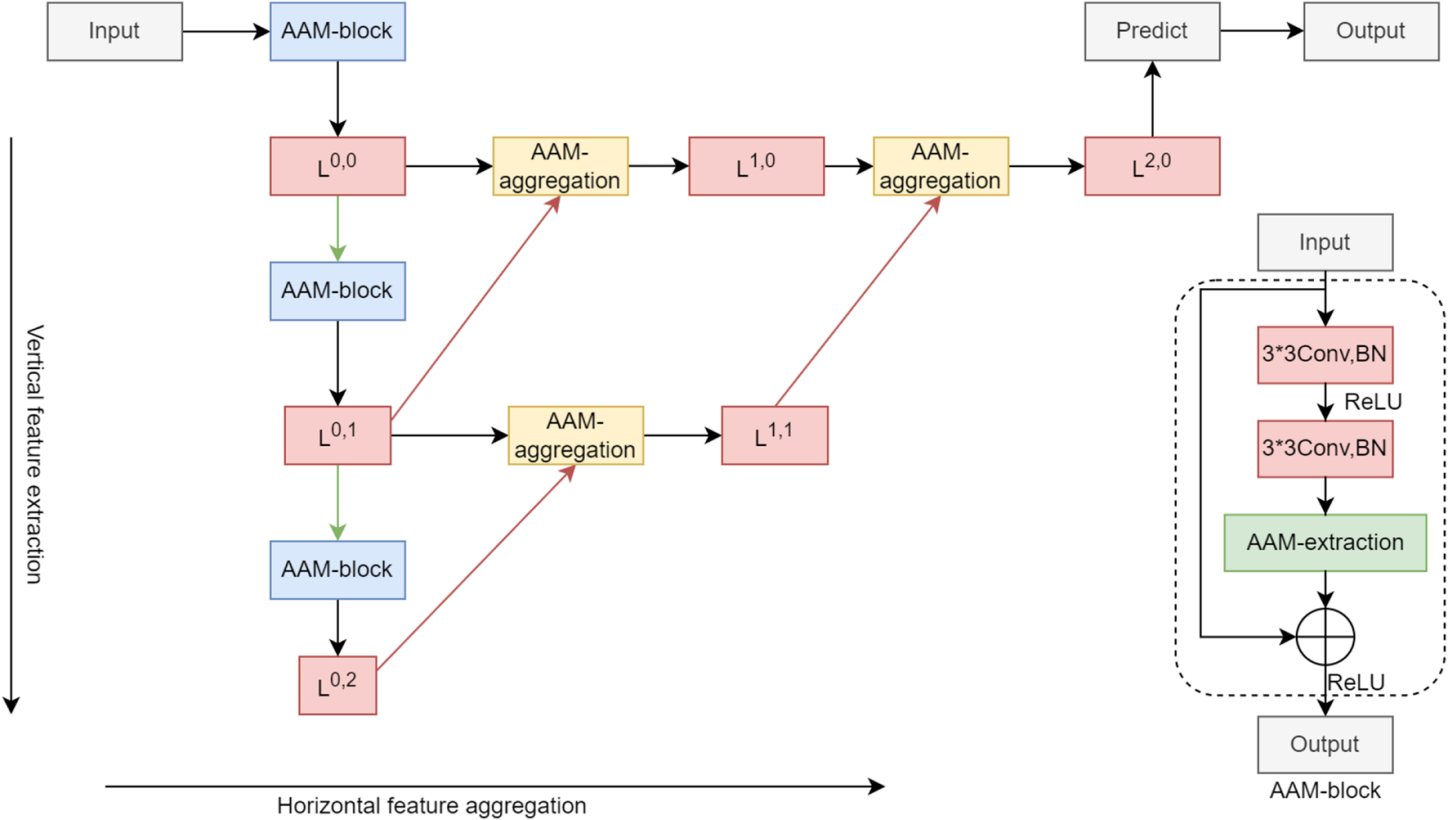
DAEA network architecture. The green and red arrows represent down-sampling and up-sampling operations, respectively. The dashed box shows the detailed flow of the AAM-block.

The network architecture is shown in Fig. 1. The input is an infrared small target image, and is passed downwards through the vertical feature extraction module, which is called the backbone network. The backbone network consists of several AAM-blocks stacked and is divided into three different stages. At each stage, high-level semantic features are extracted, and each stage is followed by a max pooling layer except the last one. Then features are propagated through the horizontal feature aggregation module. Features from neighboring stages are aggregated, with an up-sampling operation applied on the deeper feature map to match their shapes before aggregation. The resolution of the feature map is gradually restored to be the same with the input resolution. The Predict module takes the final feature map as input, and produce a binary image as output, which is the final detection result of the model.

Let $L^{i,j}$ denote the outputs of the nodes in the Fig. 1, where *i* denotes the *i*-th iteration of feature aggregation and *j* denotes the *j*-th feature extraction stage. The backbone network consists of node $L^{0,j}, j \in {0,1,2}$. The expression of $L^{i,j}$ is shown in Eq. (1).

1 $$\begin{aligned} L^{i,j} =\left\{ \begin{matrix} Ext(input) &{}i=0,j=0 \\ Ext(P_{max}(L^{i,j-1}) ) &{} i=0,j>0\\ Agg(L^{i-1,j},U(L^{i-1,j+1})) &{}otherwise \end{matrix}\right. \end{aligned}$$

where *input* is the input infrared small target image, $Ext(\cdot )$ denotes feature extraction, $Agg(\cdot )$ denotes feature aggregation, $P_{max}(\cdot )$ is the down-sampling operation using max-pooling, and $U(\cdot )$ is the up-sampling operation using bilinear interpolation.

Our network structure is similar to the U-Net^20^ structure in that both have encoding and decoding processes. However, the way of feature aggregation during decoding is different. The common approach to image segmentation is to simply aggregate shallow features with deeper features using skip connections. Our approach is to iteratively aggregate deeper features starting from the shallowest ones, while we also add attention to the process. Deep-layer feature has rich global semantic information and relatively less local detail information^23^. In infrared small target detection task, small target feature is not obvious. Hence it is important to leverage global semantic feature from deep-layers for small target recognition. Since small target feature can get overwhelmed easily in deep layer, we reduced the number of down-sampling and enhanced feature extraction by employing an asymmetric attention mechanism, we enhance feature extraction by employing an asymmetric attention mechanism, and iteratively aggregates deep and shallow features. Small target feature is continuously enhanced and the final feature map has rich global semantic information.

**Table 1:** DAEA backbone network.

Node	Output	Backbone
$L^{0,0}$	16*256*256	$$\begin{Bmatrix} 3*3conv\\ 3*3conv\\ SA \end{Bmatrix}*S$$
$L^{0,1}$	32*128*128	$$\begin{Bmatrix} 3*3conv\\ 3*3conv\\ SA \end{Bmatrix}*S$$
$L^{0,2}$	64*64*64	$$\begin{Bmatrix} 3*3conv\\ 3*3conv\\ SA \end{Bmatrix}*S$$

As shown in Fig. 1, our backbone network has blocks of cascaded convolutional layers as those in ResNet^2^. We extend residual block with an extra attention layer SA to form the AAM-block, which extracts global channel feature and local spatial detail, and uses channel shuffle to interact channel and spatial information. So the learning capability of the network is adaptively enhanced. As shown in Table 1, The down-sampling process is applied on the output of each stage except the last one, i.e. $L^{0, 0}$ and $L^{0, 1}$. The length of the backbone network can be adjusted by the hyperparameter *S*, which is the number of cascaded convolutional blocks. The number of down-sampling limits the depth of the backbone network.

The input of the horizontal aggregation node is two feature maps from preceding adjacent nodes. Because the two feature maps have different size, the deep-layer feature map is up-sampled to the same size as the shallow-layer feature map before entering the aggregation node. The aggregation node uses both global attention and local attention to extract the semantics of the high-level feature and the detail of the low-level feature, respectively. Thus, the semantic understanding of the low-level feature is enhanced and the detail deficiencies of the high-level feature are filled in. Finally, the modulated high-level and the low-level features are aggregated.

### Asymmetric attention mechanism

How to retain small target in the deep layers is the key to solve the problem of infrared small target detection. Attention mechanism is employed in the network to enhance the target feature while suppress the interference of background noise. In the field of computer vision, there are mainly channel attention and spatial attention, but it is also possible to combine both of them, e.g., Convolutional Block Attention Module (CBAM)^24^. The channel attention mechanism is a global attention that is more concerned with global semantic features and which ones are important, and the spatial attention mechanism is a local attention that is more concerned with local detail of the target and which positions need to be focused on. It is more effective to combine the two in parallel or in sequence^25^. We call such combination of global attention and local attention asymmetric attention mechanism, or AAM for short. In this paper, we applied AAM in both the feature extraction module and the feature aggregation module. AAM has two forms: self-attention in feature extraction and cross-attention in feature aggregation.

**Figure 2:**
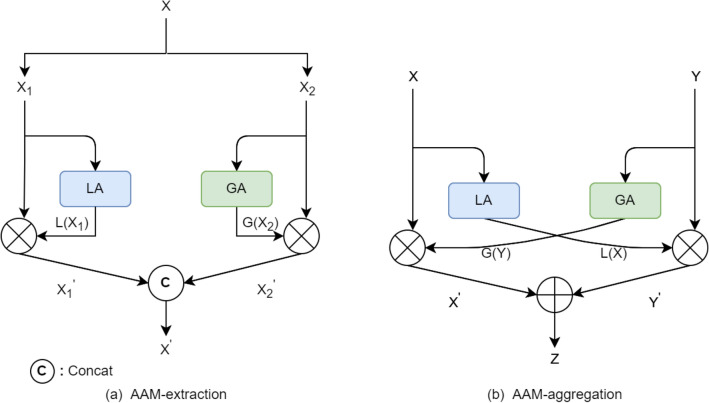
Asymmetric attention mechanism flowchart. (**a**) AAM-extraction in feature extraction process. (**b**) AAM-aggregation in feature aggregation process. GA is the global attention, LA is the local attention.

For the vertical feature extraction process, as the network goes deeper, If small targets are lost in the deep layers, then the extracted global semantic information is also invalid. Therefore, it is crucial to protect small targets on the backbone network for feature extraction. We use the self-attention form of AAM to enhance feature extraction, as shown in Fig. 2a, a global attention (GA) is used to extract semantic features, and a local attention (LA) is used to extract detail features. Both GA and LA are self-attention and applied in parallel. Then, the two branches are blended together for the two kind of features to complete each other. We call this AAM-extraction.

Similarly, in the horizontal feature aggregation process both GA and LA are applied and semantic features and detail features are blended. One difference here is that both GA and LA are in cross-attention form, as in Fig. 2b. The deep features undergo global attention to extract semantic information to enhance shallow features, and shallow features undergo local attention to extract detail information to enhance deep features, and we call this module AAM-aggregation.

#### Asymmetric attention for feature extraction

**Figure 3:**
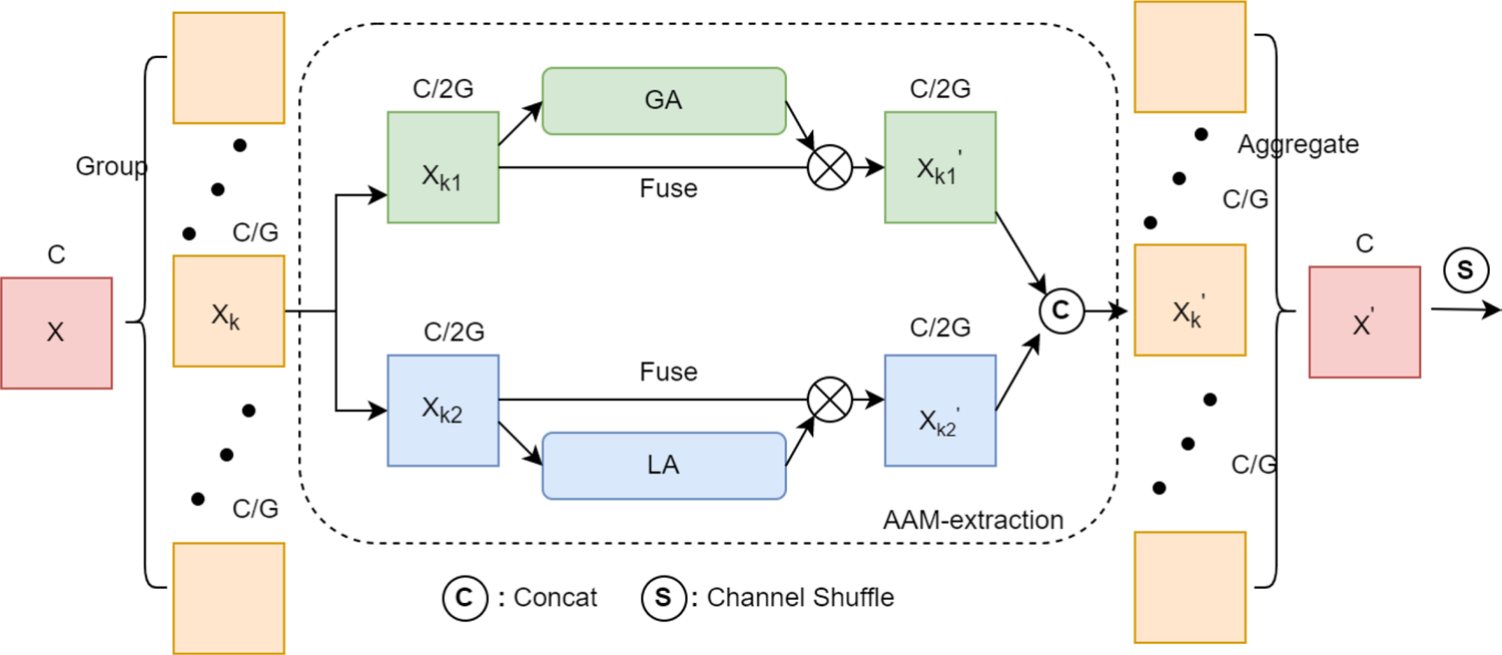
SA module flowchart. GA is the global attention, LA is the local attention.

SA is a specific implementation of AAM-extraction in the feature extraction module, which speeds up the network computation by grouping features. In this paper, we use SA as an asymmetric attention module in the vertical feature extraction process.

The overall architecture of the SA module is shown in Fig. 3. The input feature map $X\in {\mathbb {R}}^{{C}\times H\times W}$ is divided into *G* groups along the channel dimension, and each group is again divided in half along the channel dimension into sub-features $X_{k1}, X_{k2}\in {\mathbb {R}}^{\frac{C}{2G}\times H\times W}$, on which the global attention and the local attention are applied, respectively.

Specifically, the globally attended sub-feature $X_{k1}^{'}$ is produced as follows

2 $$\begin{aligned} X_{k1}^{'}=\sigma (W_{1}\cdot g(X_{k1})+b_{1})\cdot X_{k1} \end{aligned}$$

where *g* denotes global average pooling, $W_{1}, b_{1}\in {\mathbb {R}}^{\frac{C}{2G}\times 1\times 1}$ are the parameters for scaling and shifting, and $\sigma$ denotes the sigmoid function.

Similarly, the locally attended sub-feature $X_{k2}^{'}$ is given by Eq. (3).

3 $$\begin{aligned} X_{k2}^{'}=\sigma (W_{2}\cdot GN(X_{k2})+b_{2})\cdot X_{k2} \end{aligned}$$

where *GN* denotes Group Norm^26^, and $W_{2}, b_{2}\in {\mathbb {R}}^{\frac{C}{2G}\times 1\times 1 }$ are the parameters for scaling and shifting.

Then, all these attended sub-features are concatenated. And the channel shuffle operation^27^ is applied on the concatenated feature for the global and the local information to interact along the channel dimension. The model extracts both channel and spatial information of the deep-layer feature. Thus it can focus adaptively on semantic regions as well as local detail of the target, and improve the segmentation of small target significantly.

#### Asymmetric attention for feature aggregation

The global attention and local attention modules in AAM-Aggregation can be implemented in a variety of ways, and we use the global and local modules in ACM as a specific implementation of AAM-Aggregation.

Where *X* is a low-level feature map, and *Y* is a high-level feature map. Both feature maps have cross attention is used so that high-level semantic feature can attend to spatial details, and low-level feature can attend to abstract semantics.

The globally attended feature $X^{'}$ is produced by cross attention as follows

4 $$\begin{aligned} X^{'}=\sigma (\beta (w_{2}\delta (\beta (w_{1}g(Y)))))\cdot X \end{aligned}$$

where *g* denotes global average pooling, $\beta , \delta , \sigma$ denote Batch Normalization (BN), Rectified Linear Unit (ReLU), Sigmoid function, respectively, and $w_{1}\in {\mathbb {R}}^{\frac{C}{r}\times C }, w_{2}\in {\mathbb {R}}^{C\times \frac{C}{r} }$ are the parameters of two fully connected layers. The hyperparamer *r* represents the channel number reduction ratio, and 4 is used in this paper.

The locally attended feature $Y^{'}$ is produced by cross attention as follows

5 $$\begin{aligned} Y^{'}=\sigma (\beta (PWC_{2}(\delta (\beta (PWC_{1}(X))))))\cdot Y \end{aligned}$$

where $PWC_{1}$ and $PWC_{2}$ denote two point-wise convolution layers, having kernel sizes of $\frac{C}{r}\times C\times 1\times 1$ and $C\times \frac{C}{r} \times 1\times 1$, respectively. Again, *r* is the ratio of channel number reduction.

Finally, the global attention feature and local attention feature are aggregated according to $Z=X^{'}+Y^{'}$. Now, the aggregated feature map $Z\in {\mathbb {R}}^{C\times H\times W}$ is enrich with both deep semantic and spatial detail information.

## Experiment

### Loss function

As with most infrared small target detection practices, we also use the soft-IoU loss function for the network training, and the loss function is defined as Eq. (6).

6 $$\begin{aligned} L_{soft-IoU(P,L)}=\frac{ \sum _{i,j}^{}P_{i,j}\cdot L_{i,j}}{\sum _{i,j}^{}L_{i,j}+P_{i,j}-P_{i,j}\cdot L_{i,j} } \end{aligned}$$

where $P\in {\mathbb {R}}^{H\times W}$ is the prediction output of the trained network, and $L\in {\mathbb {R}}^{H\times W}$ denotes the labels.

### Evaluation metrics

Some commonly used pixel-level evaluation metrics are not applicable due to the lack of detailed textures for small infrared target. For small targets covering only a few pixels, incorrect prediction can cause a sharp drop in pixel-level evaluation metric values, so we include some metrics about the model localization ability. In this paper, the following three evaluation metrics are used to evaluate infrared small target detection.

1. Intersection over Union (*IoU*) is a pixel-level evaluation metric to evaluate the contour description capability of the algorithm by the ratio of intersecting pixels and union pixels of the predicted target and the label. The expression is shown below.
  7 $$\begin{aligned} IoU=\frac{N_{inter}}{N_{union}} \end{aligned}$$
  where $N_{inter}$ and $N_{union}$ denote the number of pixels where the predicted target intersects with the label and the number of pixels where the two are concatenated, respectively.
2. Probability of Detection ($P_{d}$) is an evaluation metric for target localization, which is the ratio of the number of correctly predicted targets to the number of all labelled targets. It indicates the capability to cover labelled targets, and a higher value means less missing targets. The expression is shown below.
  8 $$\begin{aligned} P_{d}=\frac{T_{correct}}{T_{all}} \end{aligned}$$
  where $T_{correct}$ and $T_{all}$ denote the number of correctly predicted targets and the number of all labelled targets, respectively. The correctly predicted target is defined as the target that its center-of-mass deviation is less than a given threshold. In this paper, the threshold is set to 3.
3. False Alarm Rate ($F_{a}$) is also a target-level evaluation metric. It is used to measure the ratio of false alarm pixels to all image pixels. It indicates the probability of incorrectly predicting a target, with smaller values indicating fewer incorrectly detected targets. $F_{a}$ is defined as follows
  9 $$\begin{aligned} F_{a}=\frac{P_{false}}{P_{all}} \end{aligned}$$
  where $P_{false}$ and $P_{all}$ denote the numbers of falsely predicted pixels and all image pixels, respectively. The falsely predicted pixel is defined as the centroid derivation of the target is larger than a given threshold. In this paper, the threshold is set to 3.
4. The Receiver operating characteristic curve (ROC) is used to describe the trend between the true positive rate (TPR) and the false positive rate (FPR) of a model at different thresholds, with TP, FP, TN, FN, denote true positive, false positive, true negative, false negative, in the following equation. Area Under Curve (AUC) is a quantitative indicator of ROC, with higher AUC value indicating better detection performance.
  10 $$\begin{aligned} \begin{matrix} TPR=\frac{TP}{TP+FN}&FPR=\frac{FP}{FP+TN} \end{matrix} \end{aligned}$$
  In addition, we also provide parameters (Params) and FLOPs are used to describe the complexity of the neural network. Inference time (Time) is used to indicate the speed of inference of the model.

### Implementation details

#### Datasets description

The datasets used in this experiment are NUAA-SISRT (NUAA-SISRT^5^) by Dai et al. NUDT-SISRT (^22^) by Li et al. and MDvsFA-cGAN (MDvsFA-cGAN^19^) by Wang et al. NUAA-SISRT consists of 427 infrared images and 480 instance annotations.The images in the NUAA-SISRT dataset are irregular in size, with about 55% of the targets occupying only 0.02% of the whole image, which corresponds to a target size of only 18 pixels in a 300*300 pixel image.We roughly divided the dataset into 50% training set, 30% test set and 20% validation set. The MDvsFA-cGAN dataset contains 10,000 training sets with image size of 128*128 pixels and 100 test sets with irregular image size, and we do equal division of the training set, half for training and half for validation. The NUDT-SISRT dataset contains 1,327 infrared images of small targets with image size of 256*256 pixels, and divides the dataset into 50% training set , 30% test set and 20% validation set. A large number of infrared images in the dataset have target hidden in complex background that do not stand out and are difficult to recognize even for human eye. It is difficult to solve the problem by simply setting a fixed threshold, and requires a detection network with advanced semantic understanding and the ability to retain target in deep layers.

#### Training details

Using the NUAA-SISRT, NUDT-SISRT and the MDvsFA-cGAN dataset, we conducted experiments on the PyTorch platform using a single GPU P5000-16G, CUDA 11.2. The input images are initially adjusted to a resolution of 256*256 and then normalized to all images to accelerate network convergence. Our network is trained using the soft-IoU loss function, Adagrad^28^ as the optimization method, and randomly initialized network parameters. We use a batch size of 8, an initial learning rate of 0.05. Trained 500 epochs on the NUAA-SISRT, 400 epochs on the NUDT-SISRT and 50 epochs on the MDvsFA-cGAN. The threshold value used in the predict module is 0.5.

### Comparison to the state-of-the-art methods

We compare the proposed network with several state-of-the-art (SOTA) methods. The selected model-driven methods include Top-Hat^10^, Max-Median^11^, weighted strengthened local contrast measure (WSLCM)^29^, multiscale tri-layer local contrast measure (TLLCM)^30^, Infrared patch-image (IPI)^15^, non-convex rank approximation minimization (NRAM)^31^, Reweighted infrared patch-tensor (RIPT)^16^, partial sum of the tensor nuclear norm (PSTNN)^32^, multiple subspace learning and spatial-temporal patch-tensor (MSLSTIPT)^33^. And the selected data-driven methods include U-Net^20^, Asymmetric Contextual Modulation (ACM)^5^, Attentional Local Contrast (ALC)^12^, Infrared Small-Target Detection U-Net (ISTDU)^13^ and Dense nested attention network for infrared small target detection (DNANet)^22^. The adaptive thresholds applied in the model-driven methods are calculated by the Equation 11. For the data-driven methods, we keep the same experimental parameter settings as in the respective papers.

11 $$\begin{aligned} T_{adaptive}=Max \left[ Max(G)\times 0.7,0.5\times \sigma (G)+Avg(G)\right] \end{aligned}$$

where $Max(G), Avg(G), \sigma (G)$ denotes the maximum value, the average value, and the standard deviation of the output, respectively.

#### Quantitative results

**Table 2:** Comparison of different infrared small target detection methods on the NUAA-SIRST dataset.

	Method	$IoU(\times 10^{-2})$	$P_{d}(\times 10^{-2})$	$F_{a}(\times 10^{-5})$	*Time *(*s*)	*FLOPs* (*G*)	*Params* (*M*)
Model-Driven	Top-Hat^10^	7.143	79.84	1012.00	**0.0288**	–	–
	Max-Median^11^	4.172	69.20	153.68	*0.0176*	–	–
	WSLCM^29^	1.158	77.95	625.07	14.1103	–	–
	TLLCM^30^	1.029	79.95	747.46	2.9738	–	–
	IPI^15^	25.67	85.55	11.47	0.5691	–	–
	NRAM^31^	12.16	74.52	13.85	4.2193	–	–
	RIPT^16^	11.05	79.08	22.61	1.0926	–	–
	PSTNN^32^	22.40	77.95	29.11	0.7275	–	–
	MSLSTIPT^33^	10.30	82.13	1131.00	0.083	–	–
Data-Driven	U-Net^20^	71.57	94.35	35.30	0.069	**0.31**	1.6
	ACM^5^	74.3	93.91	**7.09**	0.078	*0.30*	1.6
	ALC^12^	75.7	96.57	9.78	0.072	0.41	1.44
	DNANet^22^	**77.12**	96.33	12.77	0.064	10.91	18.7
	ISTDU-Net^13^	76.83	97.49	13.43	0.072	6.08	11.3
	DAEA (S = 3)	76.46	98.89	16.35	0.064	1.96	*1.2*
	DAEA (S = 4)	76.84	99.08	12.93	0.066	2.43	**1.6**
	DAEA (S = 5)	*77.38*	*99.44*	7.72	0.072	2.90	2.0
	DAEA (S = 6)	76.95	**99.08**	*3.53*	0.076	3.35	2.4

The best results according to each metric are marked in italic, and the second in bold.

**Table 3:** Comparison of different infrared small target detection methods on the MDvsFA-cGAN dataset.

	Method	$IoU(\times 10^{-2})$	$P_{d}(\times 10^{-2})$	$F_{a}(\times 10^{-5})$	*Time* (*s*)	*FLOPs* (*G*)	*Params* (*M*)
Model-Driven	Top-Hat^10^	4.59	61.87	151.09	**0.0288**	–	–
	Max-Median^11^	3.06	54.67	222.76	*0.0176*	–	–
	WSLCM^29^	12.73	*92.81*	259.35	14.1103	–	–
	TLCM^30^	7.61	77.69	357.62	2.9738	–	–
	IPI^15^	17.04	76.97	3.06	0.5691	–	–
	NRAM^31^	10.01	54.67	**2.56**	4.2193	–	–
	RIPT^16^	13.26	92.08	138.24	1.0926	–	–
	PSTNN^32^	16.64	69.06	3.52	0.7275	–	–
	MSLSTIPT^33^	5.12	48.92	*2.21*	0.083	–	–
Data-Driven	U-Net^20^	45.78	85.08	15.04	0.069	**0.31**	1.6
	ACM^5^	46.65	84.92	9.03	0.078	*0.30*	1.6
	ALC^12^	46.35	85.16	17.32	0.072	0.41	1.44
	DNANet^22^	43.96	78.41	10.43	0.064	10.91	18.7
	ISTDU-Net^13^	41.96	71.94	15.25	0.072	6.08	11.3
	DAEA(S=3)	47.13	85.61	23.59	0.064	1.96	*1.2*
	DAEA(S=4)	47.28	87.05	20.41	0.066	2.43	**1.6**
	DAEA(S=5)	**48.37**	86.33	10.67	0.072	2.90	2.0
	DAEA(S=6)	*48.79*	84.89	7.34	0.076	3.35	2.4

The best results according to each metric are marked in italic, and the second in bold.

**Table 4:** Comparison of different infrared small target detection methods on the NUDT-SIRST dataset.

	Method	$IoU(\times 10^{-2})$	$P_{d}(\times 10^{-2})$	$F_{a}(\times 10^{-5})$	*Time* (*s*)	*FLOPs* (*G*)	*Params* (*M*)
Model-Driven	Top-Hat^10^	20.83	78.41	107.54	**0.0288**	-	–
	Max-Median^11^	4.56	73.02	990.799	*0.0176*	-	–
	WSLCM^29^	6.82	88.47	2098.72	14.1103	–	–
	TLCM^30^	3.72	32.01	1162.42	2.9738	–	–
	IPI^15^	23.41	79.47	53.32	0.5691	–	–
	NRAM^31^	7.42	58.31	10.14	4.2193	–	–
	RIPT^16^	30.41	92.06	185.31	1.0926	–	–
	PSTNN^32^	14.87	66.98	29.06	0.7275	–	–
	MSLSTIPT^33^	12.87	62.86	24.56	0.083	–	–
Data-Driven	U-Net^20^	75.58	96.72	24.63	0.069	**0.31**	1.6
	ACM^5^	70.56	97.24	25.36	0.078	*0.30*	1.6
	ALC^12^	73.79	97.86	21.09	0.072	0.41	1.44
	DNANet^22^	*88.91*	*99.25*	*2.34*	0.064	10.91	18.7
	ISTDU-Net^13^	**86.47**	97.98	**3.71**	0.072	6.08	11.3
	DAEA(S=3)	75.37	97.52	20.61	0.064	1.96	*1.2*
	DAEA(S=4)	77.94	98.20	14.63	0.066	2.43	**1.6**
	DAEA(S=5)	81.32	**98.48**	7.23	0.072	2.90	2.0
	DAEA(S=6)	80.26	98.10	12.45	0.076	3.35	2.4

The best results according to each metric are marked in italic, and the second in bold.

The quantitative results are shown in Tables 2, 3 and  4, and the data-driven methods are more effective than the model-driven methods on all three datasets. Especially in terms of *IoU*, the model-driven methods can only reach 30.41 at best. These methods focus on target loclization, and are not good at dealing with the contour details of the target. At the same time, the manually selected parameters also limit the generalization ability of the model, which can not adapt to various complex background changes. Although several model-driven methods have achieved better $P_{d}$ results on the MDvsFA-cGAN dataset, a comparison of AUC results shows that this high detection probability is obtained with a high probability of false detection. In addition, as we can see in Fig. 5, these methods have a large number of false detections.

Compared with other data-driven methods, our model takes the shortest time to train and has the fewest parameters. For the MDvs DA-c GAN and NUAA-SIRST datasets, DAEA achieved the best results in *IoU*, $P_{d}$, and $F_{a}$. On the NUAA-SIRST dataset, our method outperforms the current SOTA with a margin of 0.26 in terms of *IoU*, and 2.87 in terms of $P_{d}$. On the MDvsFA-cGAN dataset, our method is also at the leading level. For the NUDT- SIRST dataset, which is a newly publicised dataset, our method is not yet adapted to this new dataset and is not taking the lead for now. However, our model is not far from the results of DNANet and outperforms them on the other two datasets. Therefore, in summary, our method is superior in the detection accuracy as well as shape matching of small targets.

Speaking of the parameter efficiency, with $S=3$, our method already outperforms other methods in *IoU* and $P_{d}$. We also experiment with the length of the network by tuning the hyperparameter of *S*. It demonstrates the typical *U* shape with the best performance on the NUAA-SISRT dataset when $S=5$.

**Figure 4:**
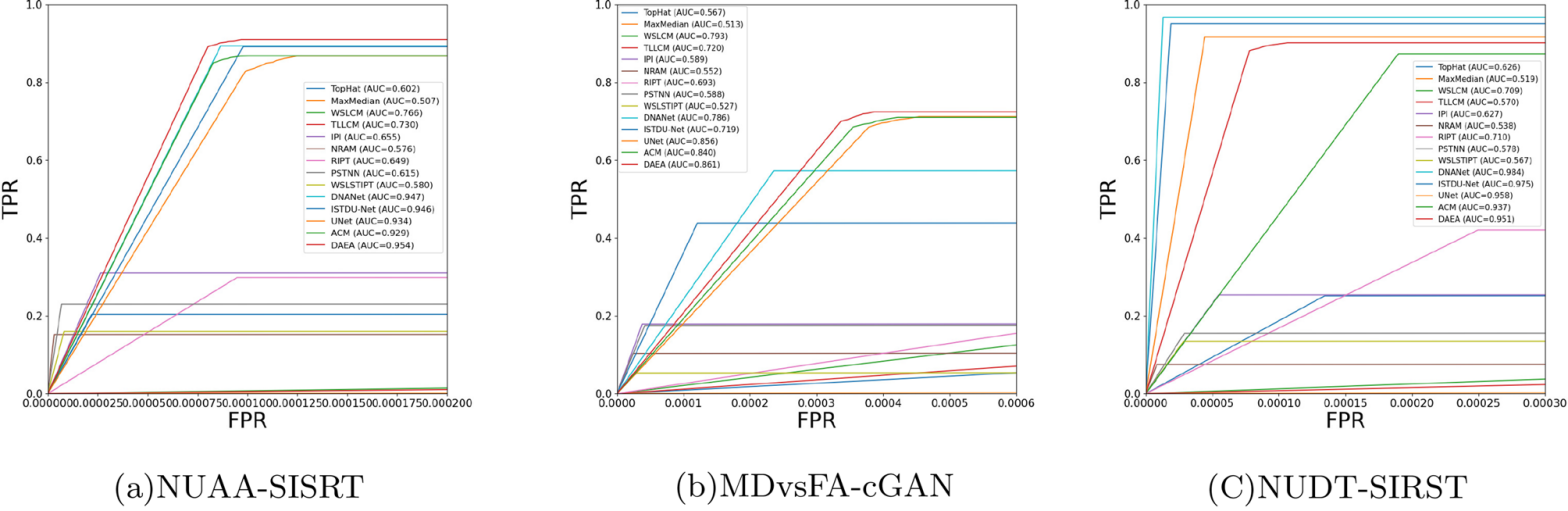
ROC curves of different infrared small target detection methods on three datasets.

As can be seen from Fig. 4, DAEA has the best AUC values on both NUAA-SIRST and MDvsFA-cGAN datasets, indicating that our method has excellent detection performance. We can also see from this that data-driven approaches generally out performs the model-driven approachs.

The images in these datasets have different complex backgrounds, target shapes and target size irregularities, which means that DAEA can learn feature that are robust to scene change.

**Figure 5:**
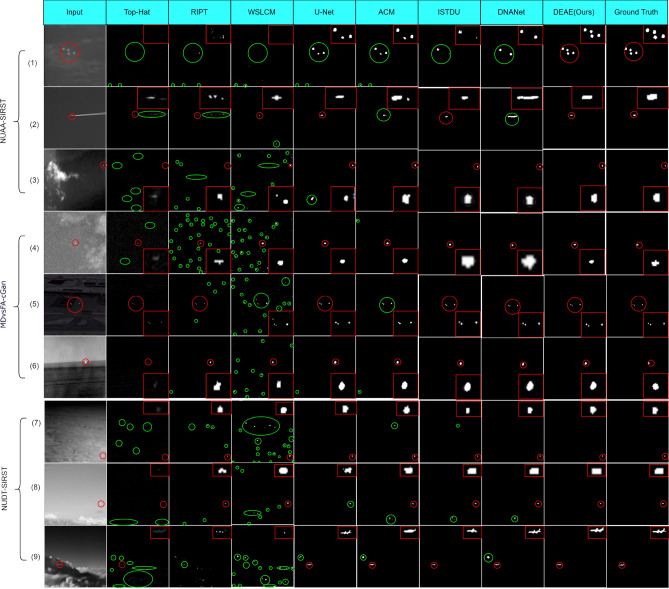
The visualization of the results achieved by different methods on 9 test images. The zoomed-in targets are shown in the red boxes. The red circles mark the areas of correctly detected targets, and the green circles mark the areas of miss targets and false alarms. Our DAEA model achieves accurate target localization as well as shape segmentation.

#### Qualitative results

**Figure 6:**
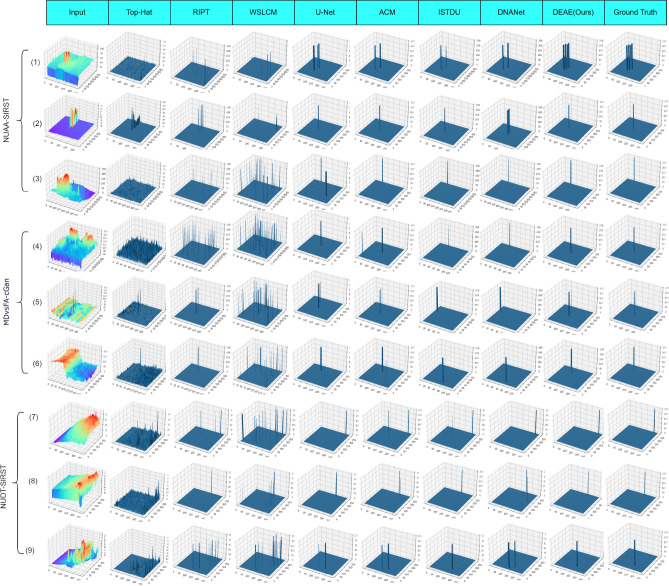
3D visualization of the results achieved by different methods on 9 test images.

Figure 5 shows the visualization of results by different methods on 9 test images, and Fig. 6 shows the 3D visualization of these results. The prediction results show that the model-driven method performs well only on the 2nd image. These methods have difficulty in distinguishing the target from the background noise with high local contrast. Hence there are a large number of miss detection and false detection, and the detected targets are displayed very faintly. This is because the features are manually selected at a shallow level, and the parameters are preset rather than learned, which result in limited generalization capability. Data-driven methods outperform model-driven methods. However, their performance are different. By comparing the results of the data-driven methods in Fig. 5, all the methods except ours have miss detection or false detection. In addition, we can see from Fig. 5(5) that DAEA has more accurate shape segmentation compared to ACM. This is because the asymmetric attention for feature extraction in the backbone network plays a key role. The small targets get retained in the deep layers by blending global and local features using asymmetric attention. The deep layer is equipped with accurate global semantic information and the global attention for feature aggregation is more effective.

### Ablation study

To investigate the role of AAM in the feature extraction and feature aggregation processes, we remove SA and ACM from DAEA, respectively. The results are shown in Table 4, from this we see that the performance of the model is severely degraded for both DAEA without SA and DAEA without ACM compared to the full-fledged model (DAEA). In particular, the *IoU* metric of the model after removing the SA module decreases by 2.74 on the NUAA-SIRST dataset, by 2.34 on the MDvsFA-cGAN dataset and by 5.11 on the NUDT-SIRST dataset, indicating that AAM plays a role in both processes in both processes. Moreover, the joint use of both AAMs in both processes works much better than using one of them alone. We replaced SA using two attentional mechanisms, Convolutional Block Attention Module (CBAM)^24^ and Squeeze-and-excitation (SE)^34^, and showed a significant decrease in model effectiveness. This suggests that it is more appropriate to use asymmetric attention mechanisms in the infrared small target detection problem.

We also investigate the advantage of asymmetry, i.e., we use symmetric attention (both with local attention or both with global attention) for both branches of the AAM. As shown in Table 5, using symmetric attention, these models do not achieve the best results, indicating the need for common attention to local detail information and global semantic information during feature extraction and feature aggregation. Asymmetric attention is more advantageous than symmetric attention.

To investigate the role of iterative aggregation in the network, we use skip-connections in U-Net instead of iterative aggregation. The results show that the effect decreases on all three datasets, with a significant rise in the particular false alarm rate, suggesting that iterative aggregation can reduce the effect of shallow noise on the predicted images.

Also we found that in the AAM-extraction module, the improvement of both LA on the model in terms of *IoU* value is greater in both datasets compared to both GA, indicating that the use of LA can help small targets to be retained on the deep feature map. In the AAM-aggregation module both GA is more effective than both LA, the *IoU* value is improved by 0.77 on the NUAA-SISRT dataset and 0.66 on the MDvsFA-cGAN dataset, which indicates that the semantic features are more important in the feature aggregation process.

**Table 5:** Results of ablation studies on asymmetric attention mechanism.

Dataset	Variant	Feature extraction	Feature aggregation	*IoU*	$P_{d} (10^{-2})$	$F_{a} (10^{-5})$
NUAA-SIRST	AAM- extraction	w/o SA	ACM	74.64	96.33	20.54
		Both GA		76.45	99.10	18.64
		Both LA		76.55	98.58	12.36
		With CBAM		76.27	98.12	15.64
		With SE		75.89	97.78	17.53
	AAM- aggregation	SA	w/o ACM	76.26	99.31	16.34
			Both GA	77.18	99.33	9.67
			Bboth LA	76.41	98.24	5.36
	U-Net DAEA	SA	ACM	76.84	98.76	25.30
				77.38	99.44	7.72
MDvsFA-cGAN	AAM- extraction	w/o SA	ACM	46.03	83.45	35.68
		Both GA		46.74	84.33	22.34
		Both LA		46.86	84.76	23.16
		With CBAM		47.35	85.33	17.63
		With SE		46.63	84.51	16.47
	AAM- aggregation	SA	w/o ACM	47.37	83.57	14.62
			Both GA	48.08	84.89	13.15
			Both LA	47.42	84.17	11.43
	U-Net DAEA	SA	ACM	47.84	85.64	16.34
				48.37	86.33	10.67
NUDT-SIRST	AAM- extraction	w/o SA	ACM	76.21	95.36	40.23
		Both GA		79.35	96.78	23.45
		Both LA		79.24	97.21	21.30
		With CBAM		80.11	97.16	15.63
		With SE		77.62	96.43	19.46
	AAM- aggregation	SA	w/o ACM	79.21	97.76	16.46
			Both GA	80.46	98.02	13.41
			Both LA	80.29	97.87	8.63
	U-Net	SA	ACM	79.21	96.23	16.79
	DAEA			81.32	98.48	7.23

## Conclusions

In this paper, we propose DAEA for the infrared small target detection problem. How to retain small target in the deep layers is the key to solve this problem. In DAEA, the asymmetric attention mechanism is used in both feature extraction and feature aggregation to get the final feature map more relevant to the small target. In the vertical flow for feature extraction, AAM-extraction is embedded into the backbone network, which can retain the feature of small target in the deep layers without being overwhelmed by the background. In the horizontal flow for feature aggregation, we use AAM-aggregation to iteratively aggregate low-level and high-level features to further highlight small target features. Better experimental results are obtained on the public datasets NUAA-SIRST, MDvsFA-cGAN and NUDT-SIRST. The ablation studies show the effectiveness and superiority of the asymmetric attention mechanism. Our future work includes exploring other forms of asymmetric attention mechanisms and making them more efficient, as well as dealing with the overfitting problem that occurs in infrared small target detection tasks.

## Data Availability

The NUAA-SISRT dataset used in this study is available at SIRST, https://github.com/YimianDai/sirst. The MDvsFA-cGAN dataset used in this study is available at MDvsFA-cGAN, https://github.com/wanghuanphd/MDvsFA_cGAN. The NUDT-SIRST dataset used in this study is available at SIRST, https://github.com/YeRen123455/Infrared-Small-Target-Detection. Source data are provided with this paper.
